# Associations of Alcohol Consumption and Chronic Diseases With Sleep Apnea Among US Adults

**DOI:** 10.5812/ijhrba.19088

**Published:** 2014-05-28

**Authors:** Yue Pan, Weize Wang, Ke-Sheng Wang

**Affiliations:** 1 Department of Public Health Sciences, Miller School of Medicine, University of Miami, Miami, USA; 2 Department of Epidemiology, Robert Stempel College of Public Health and Social Work, Florida International University, Miami, USA; 3 Department of Biostatistics and Epidemiology, College of Public Health, East Tennessee State University, Johnson City, USA

**Keywords:** Sleep Apnea Syndromes, Alcohol Drinking, Diabetes, Hypertension, Asthma, Ageing

## Abstract

**Background:**

Sleep apnea (SA) is a common sleep disorder among US adults. Associations of SA with alcohol consumption and some chronic diseases have been inconsistent.

**Objectives:**

This study aimed to estimate prevalence of SA and examine its associations with potential factors including alcohol consumption, asthma, diabetes, and hypertension.

**Patients and Methods:**

This was a cross-sectional study on 823 adults with SA and 38,638 controls from the 2011 National Survey on Drug Use and Health Data. Weighted univariate and multiple logistic regression analyses were used to examine the associations of SA with the potential factors.

**Results:**

The prevalence of SA was higher in males (4.01%) than in females (2.61%), while the prevalence increased with age (0.86%, 3.50%, and 4.47% for age groups of 18-25, 26-64, and ≥ 65, respectively). Univariate analysis revealed that all factors except for income and education were associated with SA (P < 0.05). In multivariable analyses, participants who were current and past alcohol consumers had significantly higher odds of having SA (OR = 1.52, 95% CI = 1.03-2.23; OR = 1.65, 95% CI = 1.09-2.49, respectively) than non-alcohol drinker. Furthermore, asthma (OR = 2.77, 95% CI = 2.04-3.75), diabetes (OR = 2.89, 95% CI = 2.19-3.83), and hypertension (OR = 2.42, 95% CI = 1.91-3.07) were significantly associated with SA.

**Conclusions:**

Age, alcohol consumption, asthma, diabetes, and hypertension, were positively associated with SA. More efforts should be directed to promoting screening for SA and finding possible treatments for SA among these vulnerable groups.

## 1. Background

Sleep apnea (SA) is a common sleep disorder with paused or shallow breathing during sleep (1, 2). There are three main types of SA including obstructive SA (OSA), central SA (CSA), and a mixed SA (MSA), which is a combination of OSA and CSA (3). SA has affected 1-30% of adults, depending on different study designs (4, 5). On average, about one in five adults has at least mild OSA and one of every 15 adults has at least moderate OSA (5, 6). Some found significant association between alcohol consumption and SA (6, 7), while others reported no association (8, 9). SA has been characterized as a chronic condition by high undiagnosed rate among adults as well as increasing linkage to premature death and illnesses including diabetes (10), cardiovascular disease (11, 12), obesity (12, 13), depression (14), and hypertension (15). However, the associations of SA with alcohol consumption and some chronic diseases has been inconsistent.

## 2. Objectives

The aim of this cross-sectional study was to:

1. quantify the prevalence of SA and determine how this prevalence varies with gender, age, race, marriage, income, education, alcohol consumption, and different chronic diseases such as asthma, diabetes, and hypertension in a national sample; and
2. examine the associations of SA with potential risk factors including demographics, alcohol consumption, and chronic diseases.

## 3. Patients and Methods

### 3.1. Study Population

This study used the 2011 data from the National Surveys on Drug Use and Health (NSDUH, Substance Abuse and Mental Health Services Administration (SAMHSA) (16). NSDUH is a nationally representative survey conducted annually to assess the prevalence and correlates of drug usage in the United States (US) for population aged > 12 years old. The survey provides information on the use of alcohol, tobacco, illicit drugs, and mental health problems. NSDUH survey sample is completed based on a 50-state design with an independent, multistage area probability sample for each of the 50 states and the District of Columbia. Data were collected using audio-computer or computer-assisted structured interviews. Each participant received 30$ for completing the interview. The weighted interview response rate was 74.38%. A more-detailed description of the survey procedure could be found elsewhere (16). The current analysis was restricted to adult participants aged 18 and older.

### 3.2. Measurements

#### 3.2.1. Sleep Apnea

SA was defined as dichotomized to either yes or no. Subjects were considered to have SA, if they responded “yes” to the question that whether the respondent was informed of having SA by a doctor or other medical professional in the past year.

#### 3.2.2. Demographics

Gender was self-reported as either male or female. Age was classified into three groups: 18-25, 26-64, and ≥ 65 years. Race consisted of four subgroups: white, African American (AA), Hispanic, and others. There were three categories of marital status: married; widowed, divorced or separated; never been married. The four categories of annual income were: 0-19,999$; 20,000-49,999$; 50,000-74,999$ and ≥ 75,000$. Education level was dichotomized to either less than high school or high school graduate.

#### 3.2.3. Alcohol Consumption Status

Alcohol consumption status was defined using the question “How long has it been since you last drank an alcoholic beverage?” into three categories: never (never used alcohol), current (used alcohol within the past 12 months), and past alcohol drinker (used alcohol more than 12 months ago).

#### 3.2.4. Chronic Diseases

We used asthma, diabetes, and hypertension, as the chronic disease variables. All three conditions were defined through questions asking whether doctor informed them with having the condition in past year, and were dichotomized to either yes or no.

### 3.3. Statistical Analysis

The SAS PROC SURVEYFREQ procedure was used to estimate the weighted population proportions for gender, age, race, marital status, income, education, alcohol consumption status, and chronic diseases, by the SA status. The prevalence of SA in each subgroup was reported. Chi-square test was used to compare the prevalence of SA. Then we used the SAS PROC SURVEYLOGISTIC procedure to estimate the odds ratios (ORs) and 95% confidence intervals (CIs) to assess the relation between each factor and SA. Simple logistic regressions were used to examine the independent roles of potential risk factors in SA and multiple logistic regressions were then used to simultaneously adjust all potential risk factors of SA (full model). P values < 0.05 on the two-side tests were considered statistically significant. All the analyses were performed using SAS statistical software, version 9.2 (SAS Institute, Cary, NC, USA).

## 4. Results

### 4.1. Subjects’ Characteristics and Prevalence

Table 1 shows the demographic characteristics of participants by SA case and non-SA control groups and the weighted prevalence of SA. The study sample included 823 participants who reported having SA and 38 638 controls. The prevalence of SA was higher in males (4.01%) than in females (2.61%), while the prevalence increased with age (0.86%, 3.50%, and 4.47% for age groups of 18-25, 26-64 and ≥ 65, respectively). The percentages of SA in current and past alcohol consumers were higher than non-consumers (1.86%, 3.26%, and 4.41% for non-, current and past consumers, respectively). The prevalence of SA in individuals with asthma, diabetes, and hypertension was significantly higher than in people without these diseases (7.68% vs. 3.0%, 10.64% vs. 2.71%, and 7.57% vs. 2.25%, respectively).

**Table 1.** Subjects’ Characteristics and Prevalence of SA (n = 38638) ^a, b, c^

	Number	Results	95% CI	P Value
**Gender**				< 0.0001
Male	18186	466 (4.01)	3.49-4.52	
Female	20452	357 (2.61)	2.23-2.99	
**Age group, y**				< 0.0001
18-25	18899	142 (0.86)	0.67-1.06	
26-64	17272	563 (3.50)	3.12-3.89	
≥ 65	2467	118 (4.47)	3.43-5.46	
**Race**				< 0.0001
White	24430	606 (3.91)	3.49-4.34	
AA	4934	104 (3.20)	2.32-4.08	
Hispanic	5981	69 (1.33)	0.92-1.75	
Other	3293	44 (1.37)	0.42-2.33	
**Marital status**				
Married	13627	432 (3.72)	3.24-4.19	< 0.0001
Widowed/divorced/separated	4609	187 (4.42)	3.56-5.28	
Never been married	20402	204 (1.53)	1.21-1.86	
**Income**				0.0977
0-19,999	10201	194 (3.05)	2.41-3.70	
20,000-49,999	13374	247 (2.81)	2.32-3.30	
50,000-74,999	5824	144 (3.65)	2.84-4.45	
≥ 75,000	9239	238 (3.70)	3.05-4.35	
**Education**				0.133
≤ HS	18538	356 (3.00)	2.54-3.46	
> HS	20100	467 (3.50)	3.06-3.94	
**Alcohol consumption status**				< 0.0001
Never	4961	72 (1.86)	1.23-2.49	
Current	28795	576 (3.26)	2.87-3.64	
Past	4882	175 (4.41)	3.54-5.26	
**Asthma**				< 0.0001
No	36088	697 (3.00)	2.68-3.31	
Yes	2531	126 (7.68)	5.77-9.59	
**Diabetes**				< 0.0001
No	37083	660 (2.71)	2.41-3.01	
Yes	1546	163 (10.6)	8.55-12.74	
**Hypertension**				< 0.0001
No	34490	510 (2.25)	1.96-2.54	
Yes	4126	313 (7.57)	6.45-8.68	

^a^ Abbreviations: AA, African American; HS, high school; CI, confidence interval.

^b^ Data are presented as No. (%).

^c^ P values are based on X^2^ test.

### 4.2. The Relationship between Potential Risk Factors and Sleep Apnea

The results of univariate and multiple logistic regression analyses are presented in Table 2. Univariate analysis showed that all factors except for income and education were associated with SA (P < 0.05).

**Table 2.** Univariate and Multiple Logistic Regression Analyses Assessing the Relationship Between Risk Factors and Sleep Apnea ^a^

	Crude OR	95% CI	P Value	Adjusted OR	95% CI	P Value
**Gender**						
Female	1	-	-	1	-	-
Male	1.56	1.27-1.1	< 0.0001	1.63	1.33-2.01	< 0.0001
**Age group, y**						
18-25	1	-	-	1	-	-
26-64	4.17	3.23-5.39	< 0.0001	2.10	1.51-2.92	< 0.0001
≥ 65	5.39	3.85-7.46	< 0.0001	1.76	1.15-2.69	0.0098
**Race**						
White	1	-	-	1	-	-
AA	0.81	0.60-1.10	0.182	0.82	0.59-1.14	0.239
Hispanic	0.34	0.17-0.70	0.0033	0.40	0.19-.84	0.0154
Other	0.33	0.24-.46	< 0.0001	0.42	0.30-.60	< 0.0001
**Marital status**						
Never been married	1	-	-	1	-	-
Married	2.47	1.92-3.19	< 0.0001	1.47	1.07-2.04	0.0184
Widowed/divorced /separated	2.96	2.20-3.99	< 0.0001	1.75	1.24-2.45	0.0013
**Income**						
0-19,999	1			1		
20,000-49,999	0.92	0.69-1.22	0.545	0.81	0.61-1.10	0.175
50,000-74,999	1.20	0.87-1.65	0.260	1.01	0.70-1.43	0.995
≥ 75,000	1.22	0.92-1.62	0.174	0.95	0.67-1.35	0.781
**Education**						
≤ HS	1	-	-	1	-	-
> HS	1.17	0.95-1.44	0.133	1.17	0.92-1.4845	0.204
**Alcohol consumption status**						
Never	1	-	-	1	-	-
Current	1.78	1.23-2.57	< 0.0001	1.52	1.03-2.23	0.0329
Past	2.44	1.63-3.65	< 0.0001	1.65	1.09-2.49	0.017
**Asthma**						
No	1	-	-	1	-	-
Yes	2.69	2.01-3.60	< 0.0001	2.77	2.04-3.75	< 0.0001
**Diabetes**						
No	1	-	-	1	-	-
Yes	4.27	3.33-5.47	< 0.0001	2.89	2.19-3.83	< 0.0001
**Hypertension**						
No	1	-	-	1	-	-
Yes	3.56	2.90-4.37	< 0.0001	2.42	1.91-3.07	< 0.0001

^a^ Abbreviations: CI, confidence interval; HS, high school; OR, odds ratio.

The results showed that male participants were more likely to have SA than females (OR = 1.63, 95% CI = 1.33-2.01). Compared to never married participants, married (OR = 1.47, 95% CI = 1.07-2.04) and widowed/divorced/separated ones (OR = 1.75, 95% CI = 1.24-2.45) were more likely to have SA. Our main results for the associations of SA with alcohol status and chronic diseases were consistent in both univariate and adjusted models. Participants who were current (OR = 1.52, 95% CI = 1.03-2.23) or past alcohol consumers (OR = 1.65, 95% CI = 1.09-2.49) were more likely to have SA than non-consumers. Participants who had asthma (OR = 2.77, 95% CI = 2.04-3.75), diabetes (OR = 2.89, 95% CI = 2.19-3.83) or hypertension (OR = 2.42, 95% CI = 1.91-3.07) in the past year, were significantly more likely to have SA than those without chronic disease conditions. No significant associations were found between SA and education or income in the adjusted analysis.

## 5. Discussion

In the present study, we examined the prevalence of SA and the associations of demographics, alcohol consumption, and chronic diseases with SA in a population-wide survey, NSDUH, 2011. Our study provided two main findings of interest. First, the prevalence of SA was higher in males, older ages, Whites, alcohol consumers, and individuals with asthma, diabetes or hypertension. Second, we found that alcohol consumption, asthma, diabetes, and hypertension were significantly associated with SA. Alcohol consumption has been used extensively as a sleep aid in the general population including those participants with SA or sleep disorders. It has been reported that about 28% of the insomniacs use alcohol to help them fall asleep (17). However, alcohol consumption has been demonstrated to acutely increase nasal and pharyngeal resistance (16) and may be detrimental to sleep, affect sleep stages, and worsen both insomnia and breathing disturbances during sleep (18). Epidemiological studies have not consistently demonstrated association between alcohol consumption and OSA. Some found significant associations between alcohol consumption and SA (6, 7), while others reported no association (8, 9). In our study, we reported that both current and past alcohol consumptions were significantly associated with SA. Furthermore, long-term alcohol consumption could cause obesity, a known risk factor for OSA (19).

People with asthma often have recurrent episodes of coughing, wheezing, and dyspnea during sleep (20). Several mechanisms may explain the association of asthma and SA, found in our study. Researches have suggested that airway function decreases as sleep progresses. The more a person with asthma sleeps, the greater the impairment of his/her lungs would be, leading to a more severe SA (21). Julien et al. found that asthma severity increased with a higher apnea-hypopnea index (22). A recent report from Teodorescu et al. showed that high OSA risk was associated with persistent daytime and nighttime asthma, and unrecognized OSA may be a reason for persistent asthma symptoms during the day and night (23). A recent research demonstrated that insufficient sleep was related to an increased risk for developing diabetes (24). Overall, several population and clinic-based studies have shown significantly higher prevalence of diabetes in patients with OSA (25-28). However, the results were not significant after controlling for confounders (26). Studies also showed that increased severity of OSA was associated with increased HbA1c levels and poor glucose control, after controlling the potential confounders (29, 30). In conclusion, current evidence strongly supports the association between OSA and diabetes, but a causal link remains to be examined (31). Questions of whether diabetes represents an independent risk for SA development over time or SA could cause diabetes remains to be investigated by large prospective studies.

In normal adults, sleep is associated with reduced blood pressure to about 85%-90% of the daytime level. SA has been reported to increase the blood pressure during sleep; it may lead to systemic and pulmonary hypertension and the cumulative effects could cause disruption of cardiovascular homeostatic mechanisms (32). Several epidemiological studies demonstrated that the risk of OSA was independent from hypertension (33-35). Particularly, Peppard et al. demonstrated dose-response associations between sleep-disordered breathing and hypertension in a prospective cohort study and suggested that sleep-disordered breathing was associated with an increased risk of hypertension and cardiovascular morbidity in the general population (35). However, the results were not consistent. In another cohort study, although a significant relationship between hypertension and OSA was identified, the association was diminished after controlling the BMI (36). Results from Victoria sleep cohort study found no association between OSA and incident systemic hypertension in middle-aged adults (37).

Interestingly, we also identified that being married was associated with increased risk of SA. Both currently and previously married adults had higher odds to have SA than never married ones. Previous researches showed inconsistent findings, but most reported that marriage could be a protective factor for better sleep. Grandner et al. reported that married individuals reported the least sleep complaints compared to divorced, widowed, separated, and never married people among 159 856 participants from the Behavioral Risk Factor Surveillance System (BRFSS) (38). Another study suggested that happily married middle-aged women reported fewer sleep disturbances including night awakenings, compared with women reporting lower marital happiness (39). In our study, we identified that widowed individuals had the highest prevalence of SA, but not significantly higher than the married individuals. Overall, scant researches have investigated the links between marital status and sleep. Considering the limited data available, any conclusions should be cautious.

Our study had certain limitations; first, a cross-sectional study could not reflect the causal pathway between alcohol consumption or chronic diseases and SA. A longitudinal or cohort study would be more appropriate for determining causality as well as temporal relationships of identified risk factors and SA; second, the participants who refused answering the self-report questionnaire, or were undiagnosed with SA, could cause underreporting and bias the results. Furthermore, self-reports of SA may have misclassification biases. In addition, cross-sectional study design is vulnerable to residual confounding.

Despite these limitations, our study had strengths. Previous findings of both case-control and cohort studies from biased populations could be seriously flawed by inappropriate sample sources, participation biases, and loss to follow up (5). The 2011 NSDUH is a nationally representative population-based survey with large sample size and comprehensive information for state- and local-level data, which increased the validity of our study. Well-trained staff and standardized data collection procedures provided high quality data for the analyses. Furthermore, we reported strong and significant associations of alcohol consumption and chronic diseases with SA from both univariate and multivariable regressions, adjusted for a large number of potential confounders in the model. In summary, gender, age, race, marital status, alcohol consumption, asthma, diabetes, and hypertension were all significantly associated with SA. Adults with alcohol ingestion and chronic disease conditions such as asthma, diabetes, and hypertension, may have higher risk of SA. Considering the high undiagnosed rate of SA, more efforts should be directed to promoting screening for SA as well as finding possible treatments of SA among these vulnerable groups.
